# An Untargeted Metabolomics Approach to Investigate the Metabolic Modulations of HepG2 Cells Exposed to Low Doses of Bisphenol A and 17β-Estradiol

**DOI:** 10.3389/fendo.2018.00571

**Published:** 2018-09-25

**Authors:** Nicolas J. Cabaton, Nathalie Poupin, Cécile Canlet, Marie Tremblay-Franco, Marc Audebert, Jean-Pierre Cravedi, Anne Riu, Fabien Jourdan, Daniel Zalko

**Affiliations:** ^1^Toxalim (Research Centre in Food Toxicology), Université de Toulouse, INRA, ENVT, INP-Purpan, UPS, Toulouse, France; ^2^Axiom Platform, MetaToul-MetaboHub, National Infrastructure for Metabolomics and Fluxomics, Toulouse, France

**Keywords:** HepG2, metabolomics, BPA, 17β-estradiol, endocrine disruption, metabolic network, multivariate statistics

## Abstract

The model xeno-estrogen bisphenol A (BPA) has been extensively studied over the past two decades, contributing to major advances in the field of endocrine disrupting chemicals research. Besides its well documented adverse effects on reproduction and development observed in rodents, latest studies strongly suggest that BPA disrupts several endogenous metabolic pathways, with suspected steatogenic and obesogenic effects. BPA's adverse effects on reproduction are attributed to its ability to activate estrogen receptors (ERs), but its effects on metabolism and its mechanism(s) of action at low doses are so far only marginally understood. Metabolomics based approaches are increasingly used in toxicology to investigate the biological changes induced by model toxicants and chemical mixtures, to identify markers of toxicity and biological effects. In this study, we used proton nuclear magnetic resonance (^1^H-NMR) based untargeted metabolite profiling, followed by multivariate statistics and computational analysis of metabolic networks to examine the metabolic modulation induced in human hepatic cells (HepG2) by an exposure to low and very low doses of BPA (10^−6^M, 10^−9^M, and 10^−12^M), vs. the female reference hormone 17β-estradiol (E2, 10^−9^M, 10^−12^M, and 10^−15^M). Metabolomic analysis combined to metabolic network reconstruction highlighted different mechanisms at lower doses of exposure. At the highest dose, our results evidence that BPA shares with E2 the capability to modulate several major metabolic routes that ensure cellular functions and detoxification processes, although the effects of the model xeno-estrogen and of the natural hormone can still be distinguished.

## Introduction

In the field of endocrine disrupting chemicals (EDCs), bisphenol A (BPA) is among the xeno-estrogens that have been the subject of the most extensive studies over the past two decades. In addition to its adverse effects on reproduction and development observed in rodents, BPA was found to disturb several metabolic pathways, resulting in steatogenic and obesogenic effects (1). Effects of BPA on metabolism and obesity have been assessed in several European reports. Studies carried out in rodents pre- and postnatally exposed to BPA have demonstrated significant changes in metabolic functions, evidenced by effects on lipogenesis, glucose, or insulin regulation, and body weight gain (2). Although these endpoints were not taken so far into account in the final risk characterization of BPA exposure, they have been clearly mentioned in the EFSA opinion on the risks to public health related to the presence of BPA in foodstuffs. The metabolic endpoint was not taken forward for assessing the toxicological reference point, but was taken into account in the evaluation of uncertainty for hazard and risk characterizations (2). EFSA recommended further research on the potential adverse effects of BPA for which there are uncertainties, in particular metabolic endpoints (2). In its opinion on BPA, the European Chemicals Agency (ECHA) considered it prudent to take the metabolic effects into account in hazard and risk assessment and in health impact assessment (3). In 2017, the French agency for food, environmental and occupational health and safety (ANSES) submitted a proposal to ECHA to classify BPA as a substance of very high concern within the framework of the European REACh regulation, based on its endocrine disrupting properties, including metabolic effects, which may cause serious effects to human health (4). This proposal has been adopted by ECHA's member state committee.

Although part of BPA adverse effects are attributed to its ability to activate estrogen receptors (ERs), its mechanism(s) of action at low doses remain(s) incompletely known (5). In particular, the effects of BPA on metabolism have been found to be connected with glycaemia and insulin regulation as well as lipogenesis, but little is known on the impact of BPA on biochemical mechanisms underlying observed changes in metabolic profiles, which likely involve other metabolic pathways as well (6–8).

Metabolomics based approaches are increasingly used in toxicology to investigate the biological changes induced by single toxicants or chemical mixtures, to identify markers of toxicity, and achieve a better understanding of the adverse outcome pathways (AOP) of selected chemicals (9). We previously demonstrated in CD1 mice and Sprague Dawley rats, that the metabolome is modulated in these animals following perinatal exposure to low doses of BPA (10–12). In the context of the implementation of the REACh regulation, and given the need to reduce animal experiments, *in vitro* studies become a priority in toxicity testing. High throughput assays that use cells or cell lines, preferably of human origin, are required to assess relevant disruptions in key toxicity pathways (13, 14). The development of metabolomics, and, in parallel, of bio-informatics modeling based on omics data, opens new possibilities to assess cell response to external stimuli. Combined with appropriate multivariate statistical approaches, metabolomics allows discriminating between sub-populations (of individuals or cells) according to their exposure conditions and evidencing endogenous metabolites which have significantly different levels between these groups, and therefore constitute a metabolic fingerprint of the metabolic modulations induced by the exposure. Several studies reported biomarkers discovery using such approach (15, 16).

In the present study, we used proton nuclear magnetic resonance (^1^H-NMR) based untargeted metabolite profiling followed by multivariate statistics and computational analysis of metabolic networks to examine the metabolic modulation produced in human hepatic cells (HepG2) by an exposure to low and very low doses of BPA, vs. the female reference hormone 17β-estradiol (E2). The HepG2 cell line is derived from a human hepatoblastoma. This cell line expresses biotransformation capacities for numerous xenobiotics, notably BPA (17). Expression of the estrogen receptor (ER) isoform α was established in different publications (18, 19). Specific biological effects of E2, a ligand of the estrogen receptor, were demonstrated in this cell line as well (18–21). The HepG2 cell line was selected as a cell line model broadly used in toxicology as well as metabolomic studies (16, 22, 23).

The objective of this work was to identify and compare the metabolic consequences of an exposure to low doses of the xeno-estrogen BPA and the reference hormone E2 using proton NMR metabolomics approach combined with *in silico* network reconstruction, to identify the cellular pathways most significantly modulated by these two molecules, and seek for their commonalities and differences.

## Materials and methods

### Chemicals

Bisphenol A (4,4'-isopropylidenediphenol, BPA), 17β-estradiol (E2) and dimethyl sulfoxide (DMSO) (with chemical purity > 99%) were obtained from Sigma-Aldrich (Saint Quentin Fallavier, France). Penicillin, streptomycin, trypsin, and PBS were also purchased from Sigma-Aldrich. The concentration of the stock solutions was 50 mM in DMSO.

### Cell-line

HepG2 human hepatoblastoma cells (ATCC N° HB-8065) were cultured in monolayer culture in phenol red-free αMEM (Fisher Scientific, France) supplemented with 10% fetal calf serum v/v (PAN biotech), penicillin (100 U mL^−1^), and streptomycin (100 μg mL^−1^) (Fisher Scientific), in a humidified atmosphere of 5% CO_2_ at 37°C. Continuous cultures were maintained by sub-culturing flasks every 3–5 days.

### Cells treatments and sample preparation for ^1^H NMR spectroscopy

HepG2 cells, 1 × 10^6^ cells per well, were grown in six well-plates containing 4 mL medium per well. Only one concentration was assessed per plate. Each experiment was repeated at least three times to get a final number of 18 samples per treatment. After 24 h, cells were washed in PBS and medium was replaced by serum-free and phenol red-free medium. Cells were exposed to 0.25% (v/v) DMSO in the culture medium (controls) and supplemented with BPA (10^−6^M, 10^−9^M, or 10^−12^M) or E2 (10^−9^M, 10^−12^M, or 10^−15^M) or DMSO (0.25%, control). At the end of the 24-h treatment period, cells were washed in ice cold PBS and were recovered by scraping each well twice with 1 mL ice cold water/acetonitrile (90/10, v/v). After each water/acetonitrile addition, cells were agitated with a vortex for 1 min. Samples were then centrifuged at 7000 g for 15 min at 4°C. The supernatant was then evaporated to dryness. The lyophilisates were reconstituted in 600 μL of D_2_O containing 0.25 mM TMSP (as a chemical shift reference at 0 ppm). The reconstituted solutions were transferred to NMR tubes.

### ^1^H nuclear magnetic resonance (NMR) analyses

All ^1^H-NMR spectra were obtained on a Bruker DRX-600-Avance NMR spectrometer operating at 600.13 MHz for ^1^H resonance frequency using an inverse detection 5 mm ^1^H-^13^C-^15^N cryoprobe attached to a CryoPlatform (the preamplifier cooling unit).

The ^1^H-NMR spectra were acquired at 300 K using the Carr-Purcell-Meiboom-Gill (CPMG) spin-echo pulse sequence with pre-saturation, with a total spin-echo delay (2nτ) of 320 ms to attenuate broad signals from proteins and lipoproteins. A total of 128 transients were collected into 32 k data points using a spectral width of 12 ppm, a relaxation delay of 2.5 s and an acquisition time of 2.28 s. Prior to Fourier Transformation, an exponential line broadening function of 0.3 Hz was applied to the FID.

To confirm the chemical structure of metabolites of interest, 2D ^1^H-^1^H COSY (Correlation Spectroscopy) and 2D ^1^H-^13^C-HSQC (Heteronuclear Single Quantum Coherence Spectroscopy) NMR experiments were performed on selected samples.

### Data reduction and multivariate statistical analyses to create metabolic fingerprints

Multidimensional statistical analyses of NMR data were performed using Simca-P12 software (Umetrics, Umeå, Sweden). Principal Component Analysis (PCA) was first used to detect intrinsic clusters and eventual outliers. Then Partial Least Squares—Discriminant Analysis (PLS–DA) was used to study the effect of the treatment on the cell metabolome (BPA or E_2_). This supervised method maximizes the separation between treatment groups. The number of components in the PLS models was chosen by cross validation (7-fold). The R^2^ parameter represents the explained variance. The predictive performance of the model was evaluated using the Q^2^ parameter (predictive capacity), calculated by cross-validation. Typically a robust model is characterized by a R^2^ >50% and a Q^2^ >0.4 (24).

To remove confounding variation (experimental or instrumental) not linked to the studied factor (treatment), OSC filtering was applied to the data, as such variations may overshadow the variability due to the factor under study (25). The treatment was used as a corrective factor. Filtered data were mean-centered and/or Pareto scaled.

In addition, the statistical significance and validity of the PLS-DA models were assessed using a permutation test (200 permutations). This test determines whether the specific classification of individuals in the designated groups is significantly better than any other random classification in two arbitrary groups (26).

VIP (Variable Importance in the Projection) was used to determine the most important NMR variables for the separation observed between experimental groups. VIP is a global measure of the influence of each variable on the PLS components. An arbitrary threshold of VIP >1.5 was chosen to select the variables. The Kruskal–Wallis test, a non-parametric version of Analysis of Variance, was then used to determine variables that differed significantly between groups (e.g., “discriminant” metabolites), with 0.05 being chosen as the level of significance. The set of discriminant metabolites identified from the comparison of a given treatment condition (for instance BPA 10^−6^M) vs. control conditions is considered as a “metabolic fingerprint” of the impact of this treatment exposure.

### Extraction of modulated metabolic network

Genome Scale Metabolic Networks (GSMN) aim at gathering in a single formalism all metabolic reactions which can occur in an organism (27). Each reaction is described by its substrates and products (metabolites), its stoichiometry, the enzyme(s) catalyzing it and the genes encoding the enzyme. We used the Recon2 human GSMN (28) which contains 7,440 reactions and 5,063 metabolites. In this network, metabolites are assigned to cellular compartments (mitochondria, cytoplasm…). Nevertheless, current global and untargeted metabolomics approaches do not provide information on cellular localization of metabolites. Hence, we created a modified version of Recon2 network by considering any metabolite belonging to several compartments as a single metabolite. The final modified “one-compartment” version of Recon2 that we used in our analyses contains 4,210 reactions and 2,592 metabolites.

We performed metabolic sub-network extraction from the discriminant metabolites identified from the statistical analyses. Metabolic sub-network extraction consists in computationally identifying among the 4,210 reactions, the ones that are more likely to be related to the metabolic fingerprint of each exposure. The algorithm computes the lightest path between each pair of metabolites in the fingerprint. The lightest path is a sequence of reactions and metabolites connecting two metabolites and minimizing a topological criterion in the network (29). For one metabolic fingerprint, the related sub-network is thus the union of all the lightest paths between metabolites in the fingerprint. Metabolic sub-networks were generated from the metabolic fingerprints obtained under exposure to the different doses of BPA (e.g., for instance, “BPA 10^−6^M metabolic subnetwork” for the higher dose of BPA) and E2 (for instance, the “E2 10^−9^M metabolic subnetwork”).

In order to compare the specificity of the metabolic sub-networks obtained under various exposure conditions, we compared the set of reactions and metabolites belonging to the different sub-networks. In particular, we created a sub-network specific to the effects of a BPA 10^−6^ exposure by subtracting, from the BPA 10^−6^M metabolic subnetwork, the metabolites and reactions belonging to the E2 10^−9^M metabolic sub-network. All computational and visualization tasks were performed within MetExplore web server based on the modified Recon2 metabolic network (biosource id 3223) (30, 31).

## Results

### BPA effects

**Figure 1:**
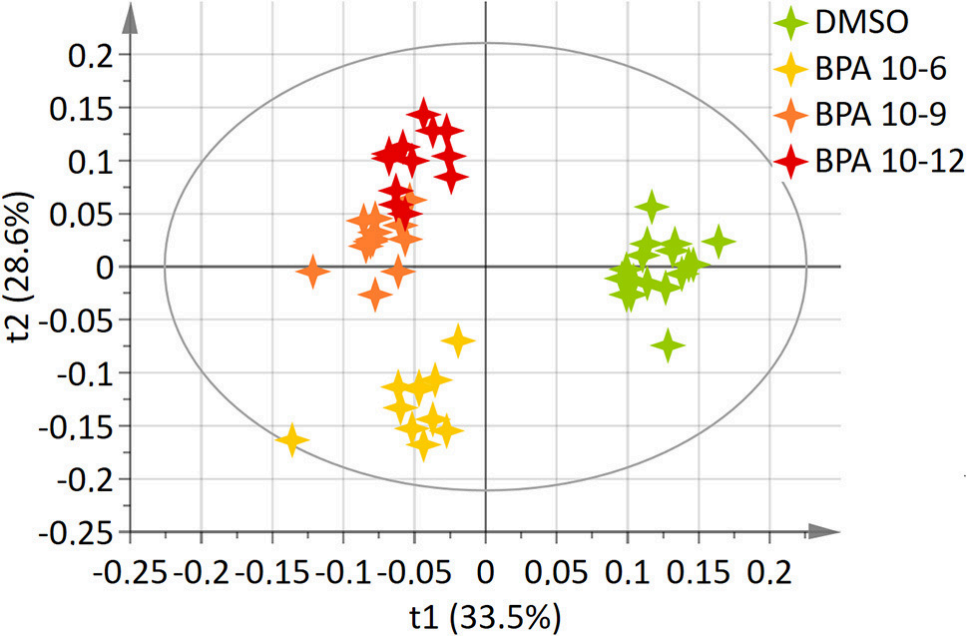
Two-dimensional PLS-DA score plot of HepG2 cell extracts integrated ^1^H-NMR spectra for BPA exposure. Each star represents an observation projected onto the first (horizontal axis) and the second (vertical axis) PLS-DA latent variables. BPA doses are shown in different colors: DMSO (green; *N* = 17), BPA 10^−6^ (light orange; *N* = 12), BPA 10^−9^ (dark orange; *N* = 12), BPA 10^−12^ (dark red; *N* = 12) (R^2^Y = 62.2% and Q^2^ = 0.561).

A two-component PLS-DA model was constructed based on all BPA-exposed groups data (4 groups: DMSO, BPA 10^−6^M, BPA 10^−9^M, and BPA 10^−12^M). This model explained 62.2% of the variability (R^2^) with a predictive ability (Q^2^ = 0.561) validating the robustness of this model (24). The score plot is presented in Figure 1, showing a clear separation between the control (DMSO) group and all BPA exposed cells along axis 1 (X axis, 1st latent variable among 2). The highest BPA dose (10^−6^M) group was discriminated from the BPA 10^−9^M and BPA 10^−12^M groups, respectively, along the 2nd axis (Y axis, 2nd latent variable). However, no significant discrimination between these two lowest BPA exposure groups (10^−9^M and 10^−12^M) was evidenced in this 4-group comparison. Twenty metabolites were found to be responsible for the separation between BPA exposed groups and the control group (Table 1). This PLS-DA model was validated by the Permutation test, confirming a robust model (32). Main metabolites involved in observed differences were amino acids, with a significant modulation of arginine, isoleucine, leucine, lysine, proline, and valine. Interestingly, specific metabolites such as glycerol, glycerophosphocholine, succinate and serine were significantly discriminant for the BPA 10^−6^M group only. Reduced glutathione was modulated in the lowest BPA dose group only, as well as AMP.

**Table 1:** Endogenous metabolite variations induced by BPA exposure (BPA samples compared to DMSO samples) in HepG2 cells.

**Metabolites**	**^1^H NMR chemical shifts (ppm)**	**BPA 10^−6^ M**	**BPA 10^−9^ M**	**BPA 10^−12^ M**
Alanine	1.48 (d,7.2); 3.79(q,7.2)		x	
AMP	4.03(m); 4.38(m); 4.51(m);6.14(d,5.9);8.27(s); 8.61(s)			x
Arginine	1.66(m); 1.74(m); 1.93(m); 3.25(t, 6.9)	x	x	x
Asparagine	2.85(dd, 16.8 and 7.4); 2.95(dd, 16.8 and 4.3); 4.01(dd, 7.4 and 4.3)			x
Citrate	2.66(d,18.1); 2.81(d,18.1)		x	x
Creatine	3.04(s); 3.93(s)	x	x	x
Dimethylglycine	2.93(s)			x
Glutamine	2.14(m); 2.46(m); 3.78(t,6.2)	x	x	x
Reduced glutathione	2.17(m); 2.56(m); 2.96(m); 3.78(m); 4.58(m)			x
Glycerol	3.57(m); 3.66(m); 3.79(m)	x		
Glycerophosphocholine	3.23(s); 3.62(m); 3.68(m);3.89(m); 3.94(m); 4.33(m)	x		
Isoleucine	0.94(t,7.4); 1.01(d,7), 1.27(m); 1.47(m); 1.98(m); 3.68(d,4)	x	x	x
Isopropanol	1.16(d,6.11); 4.01(m)		x	
Lactate	1.33(d,6.9); 4.12(q,6.9)		x	x
Leucine	0.96(t,6.3); 1.71(m); 3.74(m)	x	x	x
Lysine	1.45(m); 1.52(m); 1.73(m); 1.91(m); 3.02(t, 7.5)	x	x	x
Proline	2.01(m); 2.08(m); 2.35(m); 3.35(m); 3.42(m); 4.14(dd, 6.7 and 8.7)	x	x	
Serine	3.84(m); 3.94(dd,12.4 and 5.8); 3.98(dd, 12.4 and 3.7)	x		
Succinate	2.41(s)	x		
Valine	0.995(d,7); 1,045(d,7); 2.28(m);3,62(d,4.4)	x	x	x

*Chemical shifts (ppm) are relative to TMSP (^1^H, δ, 0 ppm). Multiplicity of signals is indicated within brackets: s, singlet; d, doublet; dd, doublet of doublet; t, triplet; q, quadruplet and m, multiplet. Values into brackets are ^1^H–^1^H splittings (Hz) in cases where these are clearly resolved. “x” represents significantly modultated concentration compared to DMSO samples*.

### 17β-estradiol (E2) effects

**Figure 2:**
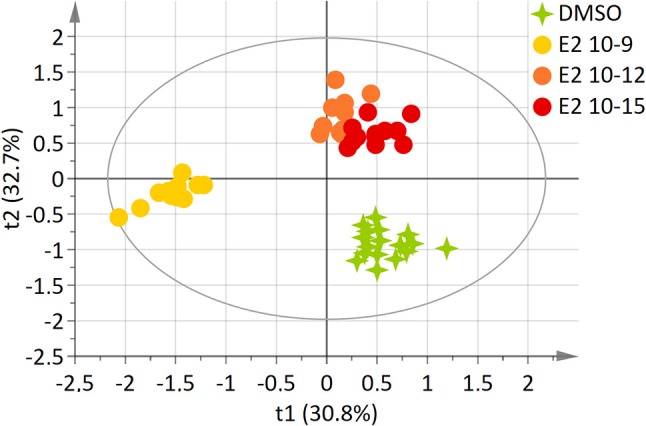
Two-dimensional PLS-DA scores plot of HepG2 cell extracts integrated ^1^H-NMR spectra for E2 exposure. Each dot or star represents an observation projected onto the first (horizontal axis) and the second (vertical axis) PLS-DA latent variables. E2 doses are shown in different colors: DMSO (green; *N* = 17), E2 10^−9^ M (light orange; *N* = 12), E2 10^−12^ M (dark orange; *N* = 12), E2 10^−15^ M (dark red; *N* = 12) (R^2^Y = 81.6% and Q^2^ = 0.635).

Using an identical approach, a two-component model was constructed for E2-exposed cells, based on the whole set of data. This model explained 81.6% of the variability (R^2^) and demonstrated a high predictive ability (Q^2^ = 0.635). The score plot showed a clear separation between the DMSO and E2 doses groups (E2 10^−12^M and E2 10^−15^M) and the E2 10^−9^M group along axis 1 (Figure 2). The “lowest” doses of E2 groups, namely E2 10^−12^M and E2 10^−15^M were discriminated from the DMSO group along the 2nd axis. No discrimination between the E2 10^−12^M and E2 10^−15^M groups was found in this four-group comparison. The PLS-DA models were validated by the permutation test (32).

Twenty-four metabolites were significantly increased or decreased and were found to be responsible for the separation between groups (Table 2). Some amino acids, namely alanine, glycine, lysine, proline, tyrosine, and valine, were identified as discriminant only for the highest dose of E2, whereas other metabolites (including acetate, formate, and isopropanol) were modulated only for the 2 lowest doses. Reduced glutathione was also modulated for the highest tested dose of E2, contrary to BPA. Choline and ethanolamine were significantly modulated at the 3 tested doses.

**Table 2:** Endogenous metabolite variations induced by E2 exposure (E2 samples compared to DMSO samples) in HepG2 cells.

**Metabolites**	**^1^H NMR chemical shifts (ppm)**	**E2 10^−9^ M**	**E2 10^−12^ M**	**E2 10^−15^ M**
Acetate	1.91(s)		x	x
Alanine	1.48 (d,7.2); 3.79(q,7.2)	x		
Asparagine	2.85(dd, 16.8 and 7.4); 2.95(dd, 16.8 and 4.3); 4.01(dd, 7.4 and 4.3)	x	x	
Choline	3.20(s); 3.52(m);4.07(m)	x	x	x
Citrate	2.66(d,18.1); 2.81(d,18.1)	x		
Ethanolamine	3.14(m); 3.82(m)	x	x	x
Formate	8.45(s)		x	x
Glucose	3.25(dd,7.3 and 7.9); 3.42(m); 3.47(m); 3.51(m); 3.54(m); 3.72(m); 3.73(m); 3.77(m); 3.84(m); 3.90(m); 4.65(d,8); 5.24(d,3.8)	x		
Glutamine	2.14(m); 2.46(m); 3.78(t,6.2)	x	x	
Glutamate	2.06(m); 2.13(m); 2.35(m); 3.77(dd,7.5 and 4.9)	x	x	
Reduced glutathione	2.17(m); 2.56(m); 2.96(m); 3.78(m); 4.58(m)	x		
Glycerol	3.57(m); 3.66(m); 3.79(m)		x	
Glycerophosphocholine	3.23(s); 3.62(m); 3.68(m);3.89(m); 3.94(m); 4.33(m)		x	x
Glycine	3.55(s)	x		
Isoleucine	0.94(t,7.4); 1.01(d,7), 1.27(m); 1.47(m); 1.98(m); 3.68(d,4)		x	x
Isopropanol	1.16(d,6.11); 4.01(m)		x	x
Lactate	1.33(d,6.9); 4.12(q,6.9)	x	x	
Lysine	1.45(m); 1.52(m); 1.73(m); 1.91(m); 3.02(t, 7.5)	x		
Phosphocholine	3.22(s); 3.58(m); 4.17(m)	x	x	
Proline	2.01(m); 2.08(m); 2.35(m); 3.35(m); 3.42(m); 4.14(dd, 6.7 and 8.7)	x		
Serine	3.84(m); 3.94(dd,12.4 and 5.8); 3.98(dd, 12.4 and 3.7)		x	
Succinate	2.41(s)		x	
Tyrosine	3.09(dd,14.5 and 7.5); 3.21(dd,14.5 and 5,1); 3.95(dd,7.5 and 5.1); 6.9(d,8.5); 7.20(d,8.5)	x		
Valine	0.995(d,7); 1,045(d,7); 2.28(m);3,62(d,4.4)	x		

*Chemical shifts (ppm) are relative to TMSP (^1^H, δ, 0 ppm). Multiplicity of signals is indicated within brackets: s, singlet; d, doublet; dd, doublet of doublet; t, triplet; q, quadruplet and m, multiplet. Values into brackets are ^1^H–^1^H splittings (Hz) in cases where these are clearly resolved. “x” represents significantly modultated concentration compared to DMSO samples*.

### Comparison of BPA and E_2_ effects

**Figure 3:**
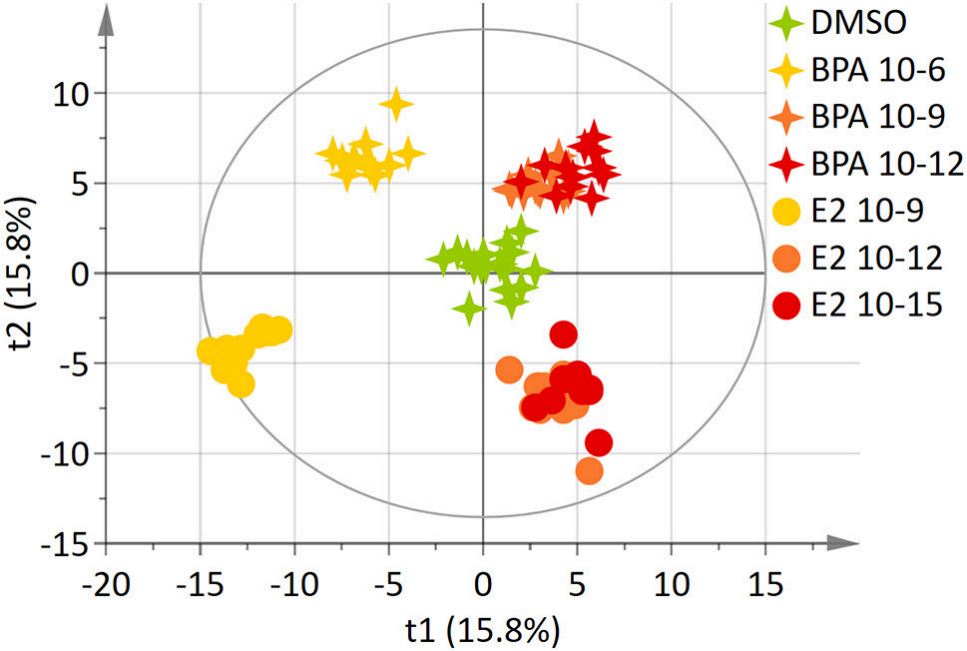
Two-dimensional PLS-DA scores plot (Axis 1 and 2) of HepG2 cell extracts integrated ^1^H-NMR spectra for BPA and E2 exposure. Each dot or star represents an observation projected onto the first (horizontal axis) and the second (vertical axis) PLS-DA latent variables. Different symbols are used for BPA (4-point star) and E2 (circle) exposure. Doses are shown as follow: DMSO: green (*N* = 17), BPA 10^−6^M: light orange (*N* = 11), BPA 10^−9^M: dark orange; (*N* = 12), BPA 10^−12^M: dark red (*N* = 12), E2 10^−9^M: light orange (*N* = 11), E2 10^−12^M: dark orange (*N* = 12), E2 10^−15^M: dark red (*N* = 12); (R^2^Y = 59.5% and Q^2^ = 0.508).

The 7-group comparison generated a 4-component PLS-DA model, explaining 59.5% of the treatment variability and having a Q^2^ value of 0.508 (robust model). Figure 3 displays the projection of the 2 first latent variables (out of 4), demonstrating a marked separation between the effects of “low” estrogenic doses (BPA 10^−9^M, BPA 10^−12^M, E2 10^−12^M, and E2 10^−15^M) and that of higher doses (BPA 10^−6^M and E2 10^−9^M), along axis 1. In addition, a marked discrimination between the effects of the xeno-estrogenic model compound BPA and that of the natural hormone E2 was observed along axis 2. All these groups were clearly separated from the DMSO (control) group. This 7-group comparison model was further validated by the permutation test step. As detailed in Table 3, 19 metabolites were found to be discriminant between the 6 exposed groups and the control group (DSMO), respectively. Some of these discriminant metabolites, for instance arginine and glutamine, were found to be similar for all BPA and E2 exposed groups, whatever the dose. Conversely, the modulation of metabolites such as creatinine, citrate and leucine (BPA, all doses) and ethanolamine (E2, all doses) appeared to be molecule specific.

**Table 3:** Endogenous metabolite variations induced by BPA or E2 exposure (BPA or E2 samples compared to DMSO samples) in HepG2 cells.

**Metabolites**	**^1^H NMR Chemical shifts δ (ppm)**	**BPA 10^−6^M**	**BPA 10^−9^ M**	**BPA 10^−12^ M**	**E2 10^−9^ M**	**E2 10^−12^ M**	**E2 10^−15^ M**
Arginine	1.66(m); 1.74(m); 1.93(m); 3.25(t, 6.9)	x	x	x	x	x	x
Asparagine	2.85(dd, 16.8 and 7.4); 2.95(dd, 16.8 and 4.3); 4.01(dd, 7.4 and 4.3)				x		
Choline	3.20(s); 3.52(m);4.07(m)				x		
Citrate	2.66(d,18.1); 2.81(d,18.1)	x		x			
Creatine	3.04(s); 3.93(s)	x	x	x			
Ethanolamine	3.14(m); 3.82(m)				x	x	x
Glutamate	2.06(m); 2.13(m); 2.35(m); 3.77(dd,7.5 and 4.9)				x	x	
Glutamine	2.14(m); 2.46(m); 3.78(t,6.2)	x	x	x	x	x	x
Reduced glutathione	2.17(m); 2.56(m); 2.96(m); 3.78(m); 4.58(m)			x	x		
Glycero phosphocholine	3.23(s); 3.62(m); 3.68(m);3.89(m); 3.94(m); 4.33(m)					x	x
glycine	3.55(s)				x		
Isoleucine	0.94(t,7.4); 1.01(d,7), 1.27(m); 1.47(m); 1.98(m); 3.68(d,4)	x	x	x			x
Leucine	0.96(t,6.3); 1.71(m); 3.74(m)	x	x	x			
Lysine	1.45(m); 1.52(m); 1.73(m); 1.91(m); 3.02(t, 7.5)	x	x	x	x	x	
Phosphocholine	3.22(s); 3.58(m); 4.17(m)				x	x	
Proline	2.01(m); 2.08(m); 2.35(m); 3.35(m); 3.42(m); 4.14(dd, 6.7 and 8.7)	x	x		x		
Succinate	2.41(s)				x	x	
Tyrosine	3.09(dd,14.5 and 7.5); 3.21(dd,14.5 and 5,1); 3.95(dd,7.5 and 5.1); 6.9(d,8.5); 7.20(d,8.5)			x	x		
Valine	0.995(d,7); 1,045(d,7); 2.28(m);3,62(d,4.4)	x	x	x	x		

*Chemical shifts (ppm) are relative to TMSP (^1^H, δ, 0 ppm). Multiplicity of signals is indicated within brackets: s, singlet; d, doublet; dd, doublet of doublet t, triplet; q, quadruplet and m, multiplet. Values within brackets are ^1^H–^1^H splittings (Hz) in cases where these are clearly resolved. “x” represents significantly modulated concentration compared to DMSO samples*.

Finally, we proceeded to network analysis based on ^1^H-NMR data, to further investigate the effects shared by the two molecules.

### Network of BPA and E2 effects

**Figure 4:**
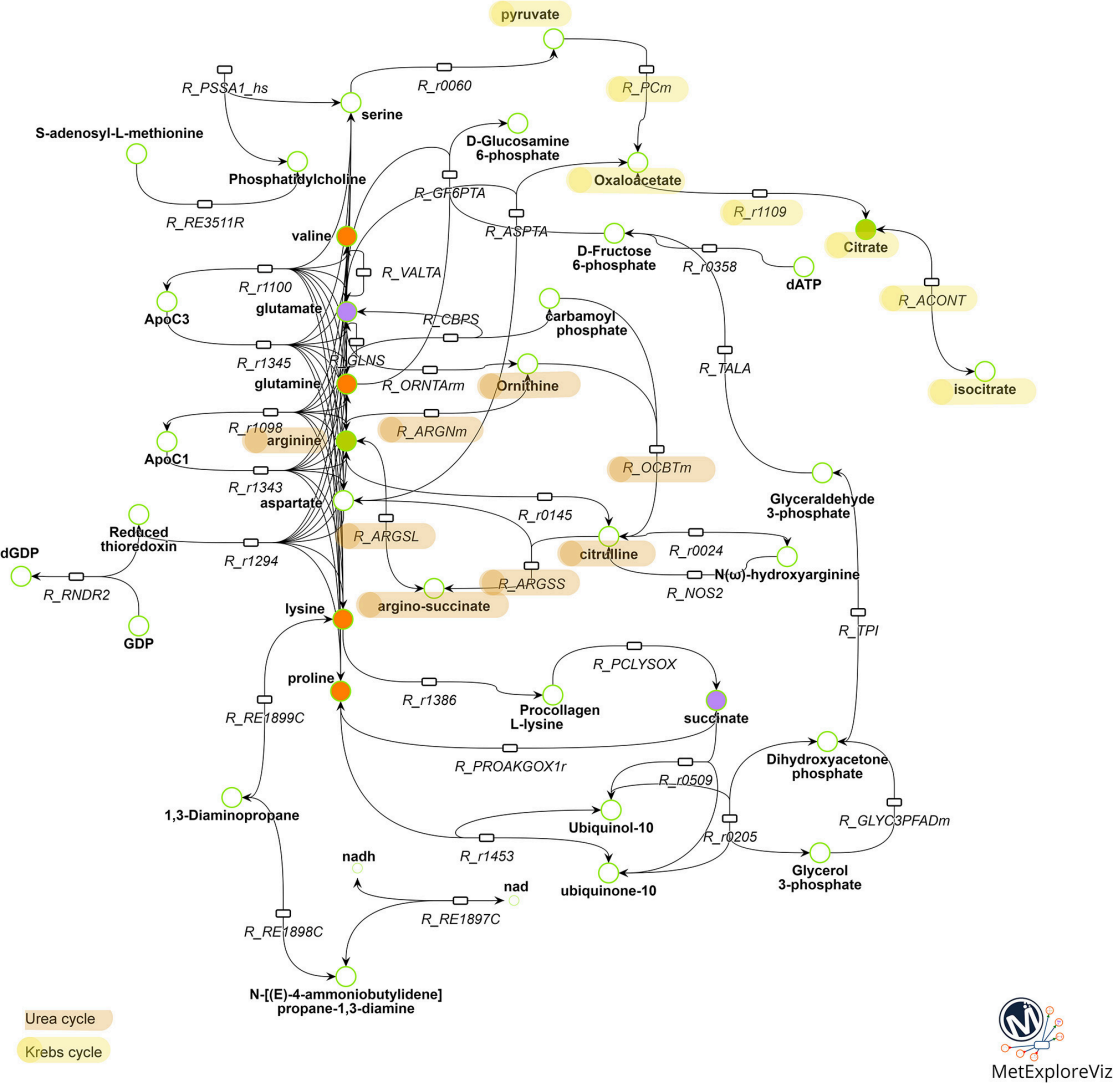
Metabolic pathways commonly modulated by BPA 10^−6^M and E2 10^−9^M exposure. Reactions and metabolites belonging to both the BPA 10^−6^M and E2 10^−9^M metabolic sub-networks are represented. Reactions are represented by rectangles and metabolites by circles. Metabolites identified as discriminant in the BPA 10^−6^M (resp. E2 10^−9^M) group compared to the control group according to the PLS-DA model are colored in green (resp. purple). Metabolites identified as discriminant in the both BPA 10^−6^M and E2 10^−9^M groups are colored in orange. Reactions corresponding to biologically non-relevant paths between metabolites were filtered out. For clarity, substrates and products of the reactions, not specifically belonging to the BPA 10^−6^M and E2 10^−9^M metabolic sub-networks, are not represented. The whole list of reaction and metabolite names is presented in Supplemental Table 3.

Multivariate analysis revealed that BPA 10^−6^M and E2 10^−9^M appear to share some metabolic effects (axis 2) but also have specific effects (axis 1), suggesting both common and molecule-specific mechanisms of action. In order to further distinguish between shared mechanisms (which likely reflect an estrogenic effect) from the effects specific to BPA 10^−6^M, we performed a network analysis. Two networks were reconstructed based on metabolic fingerprints obtained in the 7-group analysis (see Supplemental Figures 1, 2, Supplemental Tables 1, 2, and the Material and Methods section for sub-network extraction). We extracted the common part for these two subnetworks, resulting in a common BPA-E2 sub-network (Figure 4). This sub-network strongly suggests that the common target between BPA and E2, as regards metabolome modulation, relies on the modulation of metabolic pathways involving specific amino acids, namely valine, proline, lysine, and glutamine and, notably, the pathways involving the production/degradation of apolipoprotein C3 (ApoC3) and apolipoprotein C1 (ApoC1). Other key biochemical pathways likely modulated both by BPA and E2 high exposure doses were identified following network reconstruction, including metabolites and reactions involved in the urea cycle (citrulline, arginine, argino-succinate, and ornithine) and in the Krebs cycle (isocitrate, citrate, oxaloacetate, pyruvate).

Finally, with the aim to explore the specific effects of BPA, we subtracted from the BPA network the part shared with E2. In accordance with the metabolomic data that evidenced isoleucine and leucine as specifically discriminant in BPA-exposed groups (but not in E2 exposed groups), these two amino acids were highlighted as major endogenous metabolites modulated by BPA in the BPA-specific subnetwork. Network reconstruction also identified some intermediates in the metabolism of leucine and isoleucine, namely isovaleryl CoA and methylbutanoyl-CoA, that are therefore likely to be specifically modulated by BPA exposure.

## Discussion

In this study, we used a proton NMR global metabolomics approach, with no *a priori*, to compare the effects of a range of concentrations of E2 and BPA on a broadly used human hepatic cell line (HepG2). Then, we used bioinformatics (Metabolic Network modeling) methods to further investigate the biological pathways modulated by these estrogens: E2 as the reference hormone, and BPA as a model xeno-estrogen with well-documented endocrine disrupting properties, but also strongly suspected metabolic effects. Many EDCs with xeno-estrogenic properties can act as metabolic modulators (1, 33, 34). We previously demonstrated metabolic changes in the liver and brain of mice exposed during *intra-utero* development and lactation, to low doses of BPA (10). However, assessing the effects of EDCs using metabolomics approaches requires substantial resources, especially *in vivo*. The number of chemicals that need to be assessed as regards their potential to induce metabolic changes, and the necessity to comply with the “Three Rs” (3Rs) guiding principles for more ethical use of animals in testing, require developing alternative *in vitro* bioassays, preferably based on human models. The implementation of metabolomics and bioinformatics on the bases of *in vitro* bioassays first needs to be validated with model molecules. It also opens the road for a better understanding of the mechanisms of action of EDCs. These approaches should be as sensitive and relevant as possible to further examine the reliability of biomarkers of effects of EDCs. Experiments carried out to seek for these biomarkers should also link to low, environmentally relevant, exposure levels of EDCs in human body, as mentioned for xeno-estrogens by Wang et al. regarding BPA (35).

The HepG2 cell line, derived from a human hepatoblastoma, was selected to perform this work as it is broadly used in toxicology, including in studies combining *in vitro* cell systems and metabolomics for the identification of the mode of action (MoA) (16, 22). Although this cell line is of limited metabolic capacity compared to human hepatocytes, it has been demonstrated to be efficient in the biotransformation of BPA and the metabolic pathways were found to be similar to those observed in humans (17, 36, 37). Expression of the estrogen receptor (ER) isoform α was established in different publications (18, 38). Likewise, specific biological effects of 17β-Estradiol (E2), a ligand of the estrogen receptors, were demonstrated in this cell line (18–21, 38). These cells were exposed to environmental relevant concentrations of BPA and E2. The selected doses (10^−6^M, 10^−9^M, and 10^−12^M for BPA, and 10^−9^M, 10^−12^M, and 10^−15^M for E2) were in accordance with the bibliography regarding their respective estrogenic potency, as it is estimated that the estrogenic potency of E2 is approximately 1 000 to 10 000 time greater that BPA' s estrogenic potency *in vitro* (39, 40).

Although few papers already reported the effects of low doses of BPA exposure on HepG2 cells, these studies mainly used targeted approaches, providing results on hepatotoxic endpoints, such as serum aspartate aminotransferase (AST) and alanine transferase (ALT) measurements, or inflammatory genes analyzed by RT-qPCR (41, 42). In our study, we applied non targeted ^1^H-NMR metabolomics approach to HepG2 cells extracts to evidence the impact of BPA on the whole metabolism, in order to gain a more “global picture” and with no *a priori* hypothesis regarding the effect of this endocrine disruptor. The same methodology was applied to estradiol as the reference estrogenic compound, to identify common and specific metabolic pathways modulated by both molecules, with the benefit of bioinformatics and network reconstruction to help identifying metabolites not detected directly during analyses, but yet playing a role in the metabolic pathways modulated by BPA and/or E2.

Several studies have reported that BPA and E2 are acting the same way *in vitro*. For instance, BPA and E2 were both shown to promote HepG2 cell proliferation by inhibition of apoptosis and stimulation of telomerase activity via an estrogen receptor-dependent pathway (43). In our study, metabolomics revealed modulations of the HepG2 metabolome that are molecule specific (discrimination between the BPA exposed groups and the E2 exposed group). Although some metabolic pathways are common to BPA and E2, it is interesting to note that parts of the metabolic fingerprints are different between the 2 molecules at the highest tested concentration (10^−6^M for BPA and 10^−9^M for E2, respectively). This specific concentration of BPA was reported to be estrogenic *in vitro* in several models such as MCF-7 cells and ZELH-zfERs cell lines (44, 45). However, our results suggest that BPA is able to induce a metabolic modulatory effect, even at higher and estrogenic doses, which is different from the metabolic modulation at the estrogenic dose of E2 (10^−9^M). According to Gould et al., BPA interacts with the ERα in a distinct manner from estradiol. BPA is not merely a weak estrogen mimic but exhibits a distinct mechanism of action at the level of ERα. The distinct activity of BPA is most likely due to an induction of a conformation of the activated ERα by BPA that differs from these other known classes of ER ligands. Generally, BPA is considered as a weak estrogenic compound (46). Nevertheless, and despite its relatively low affinity for ERα, an increasing number of studies have demonstrated that BPA can promote estrogen-like activities that are similar (or even stronger) than the ones elicited by 17β-estradiol (47). These low-dose responses result in part from the activation of rapid responses via non-classical ER pathways or by a different BPA recruitment of co-activators or co-repressors (48, 49). For instance, low doses of BPA only induce gene expression related to lipid synthesis and trigger triglyceride accumulation in adult mouse liver (8).

Metabolomics has already been applied to *in vivo* samples from mice and rats. In both cases, we already revealed modulations of energy metabolism (10, 12), which was also observed in other organisms such as Daphnia magna, together with a modulation of part of the same amino acids (such as arginine, glutamine, lysine, valine), and lactate (50). In our case, we also observed changes in many amino acids, as well as in metabolites involved in energy metabolism. The modulation of cholines suggest a modulation in the lipids composition in the cell and in the membrane fluidity at the estrogenic dose (10^−6^M BPA), which is not the case for the two other BPA doses tested. Interestingly, reduced glutathione was modulated in the lowest BPA dose group only, which may suggest a change in the capacity of detoxification of the cells through this specific biochemical pathway. More importantly, differences in reduced glutathione levels also determine the expressed mode of cell death, being either apoptosis or cell necrosis. Lower levels of reduced glutathione may result in the systematic breakage of the cell which may lead to cell death (51). The metabolic network reconstruction highlighted paths of metabolic reactions specifically modulated by BPA exposure but not by E2 exposure. These paths include leucine and isoleucine, but also intermediary metabolites of the metabolism of these 2 amino acids (isovaleryl-coenzyme A and 2-methylbutanoyl-CoA), suggesting that the metabolism of branched-chain amino acids might be modulated by BPA with possible consequences in the promotion of protein synthesis and turnover, signaling pathways, and the metabolism of glucose (52). Isoleucine, like the other branched-chain amino acids, is associated with insulin resistance: higher levels of isoleucine are observed in the blood of diabetic mice, rats, and humans (53). Also, oxidation of such amino acids may increase fatty acid oxidation and play a role in obesity, which is consistent with the fact that BPA is a candidate “obesogen” (54).

Regarding the specific identification of the commonly modulated parts of the BPA and E2 metabolic networks, it is interesting to note the presence of the urea cycle, including metabolites such as citrulline, ornithine and argino-succinate, not evidenced by the metabolomic analyses but highlighted by the metabolic network comparison. This finding is in agreement with published data on a subset of ToxCast chemicals including BPA (55).

Another interesting outcome of the metabolic network analysis is the presence of apolipoprotein C1 (ApoC1) and apolipoprotein C3 (ApoC3) pathways in the shared mechanism between E2 and BPA. These components of high density lipoproteins (HDL) and very low density lipoproteins (VLDL) respectively, are in charge of the uptake, transport and catabolism of lipids. It has already been reported that ApoC3 was affected by BPA exposure, as is the expression of the APOC3 gene, which was found to be decreased in the liver of BPA-exposed C57BL/6 mice (56, 57). It was also reported that BPA modulates novel binding sites for SREBP-1 in genes directly or indirectly involved in cholesterol metabolism, such as APOC3(57). BPA exposure was also reported to be driving the upregulation of SREBP-1 and SREBP-2 *in vivo* (C57/Bl6 mice) and *in vitro* (HepG2 and Caco cells) (56, 58). In intestinal cells, SREBP-2 may be involved in the BPA-induced cholesterol absorption, leading consequently to hypercholesterolaemia (58). Some metabolites (isocitrate, oxaloacetate, and pyruvate) and reactions (citrate synthase, aconitase, and pyruvate carboxylase) involved in energy metabolism, and more specifically in the first steps of the Krebs cycle, were also pointed out as a potential common target for BPA and E2 according to the network analysis. The Krebs cycle was already identified *in vivo* after a perinatal exposure of CD1 mice, as well as for a longitudinal study in Sprague Dawley rats exposed perinatally, all of them to low doses of BPA (10, 12, 35). Although it will be necessary to confirm these *in silico* suggestions by more targeted biochemical assays, these results once again reinforce the fact that BPA and E2 may be considered as metabolic modulator chemicals that are interfering with the energy metabolism.

Network analysis allows identifying reactions that are likely to be modulated by the various exposure conditions. One of the key challenge in metabolic network analysis is the quality of the metabolic network reconstruction used. In fact, since these networks are built based on genomic information they may contain false positive reactions (reactions which should not be included) and false negatives (missing reactions). Recon2 is a highly curated reconstruction for the human metabolic network applied in many studies, but improved versions are regularly released, such as Recon3D, which has been recently introduced but has still not been used in the field of metabolomics (59–62). The other challenge is the algorithm choice to extract sub-networks. As discussed in Frainay and Jourdan (2017), there are several options to extract paths connecting metabolites and their efficiency largely depends on the application: the lightest path option, chosen here, proved to be specifically relevant and efficient to be used in metabolomics (29).

To go further with these findings, it would be interesting to perform non-targeted lipidomics to identify which family of lipids are modulated by BPA and to complete these identifications using targeted lipidomics for specific lipid families. To complete our metabolomics set of data, and to improve the quality of the metabolic networks, mass spectrometry metabolomics could be performed in order to get access to a larger set of discriminant metabolites in complement of the NMR generated list.

In summary, our study highlights that BPA, not only behaves as a xeno-estrogen as regards its potential to impact fertility and reproduction, but also shares with the natural hormone E2, the capability to modulate major metabolic routes that ensure cellular functioning and detoxification processes. We also evidenced that BPA and E2 both exert distinct effects at low and high concentrations in HepG2 cells and may act through different mechanisms. This is consistent with many reports about the non-monotonic dose effects of BPA and with our current understanding of the functioning of natural hormones, which can trigger different responses in the organism, depending on their circulating level (34, 63). Moreover, the results of this study, consistent with our previous *in vivo* results, provide first proofs of evidence that metabolomics combined with network reconstruction can be used *in vitro* (here on the human HepG2 model) as relevant approaches to investigate commonalities and differences in MoA. An even greater added value of metabolic network will come with a metabolite list as complete and specific as possible to identify the most impacted pathways associated with BPA exposure. Connecting these biochemical pathways with MoA will help to identify AOP and to facilitate the hazard characterization of other compounds (61).

## Author contributions

NC and NP contributed to synthesize and interpret the data, and writing this manuscript (MS). MA contributed to supervising the cell work to generate the data, and contributed to revising the manuscript. CC was responsible of the NMR analyses and contributed in the writing of this specific section. MT-F was in charge of the statistical analyses and contributed to the writing of this section. J-PC contributed to the writing of this MS, AR contributed in supervising the experiments and revising this MS, FJ contributed in performing the metabolic networks and the writing of the MS. DZ contributed in supervising all this work, including the writing of this MS.

### Conflict of interest statement

The authors declare that the research was conducted in the absence of any commercial or financial relationships that could be construed as a potential conflict of interest.
