# Supplementary figures and images for: An Untargeted Metabolomics Approach to Investigate the Metabolic Modulations of HepG2 Cells Exposed to Low Doses of Bisphenol A and 17β-Estradiol
Source: Front Endocrinol (Lausanne). 2018 Sep 25;9:571. doi: 10.3389/fendo.2018.00571 (PMC6167423; doi:10.3389/fendo.2018.00571)

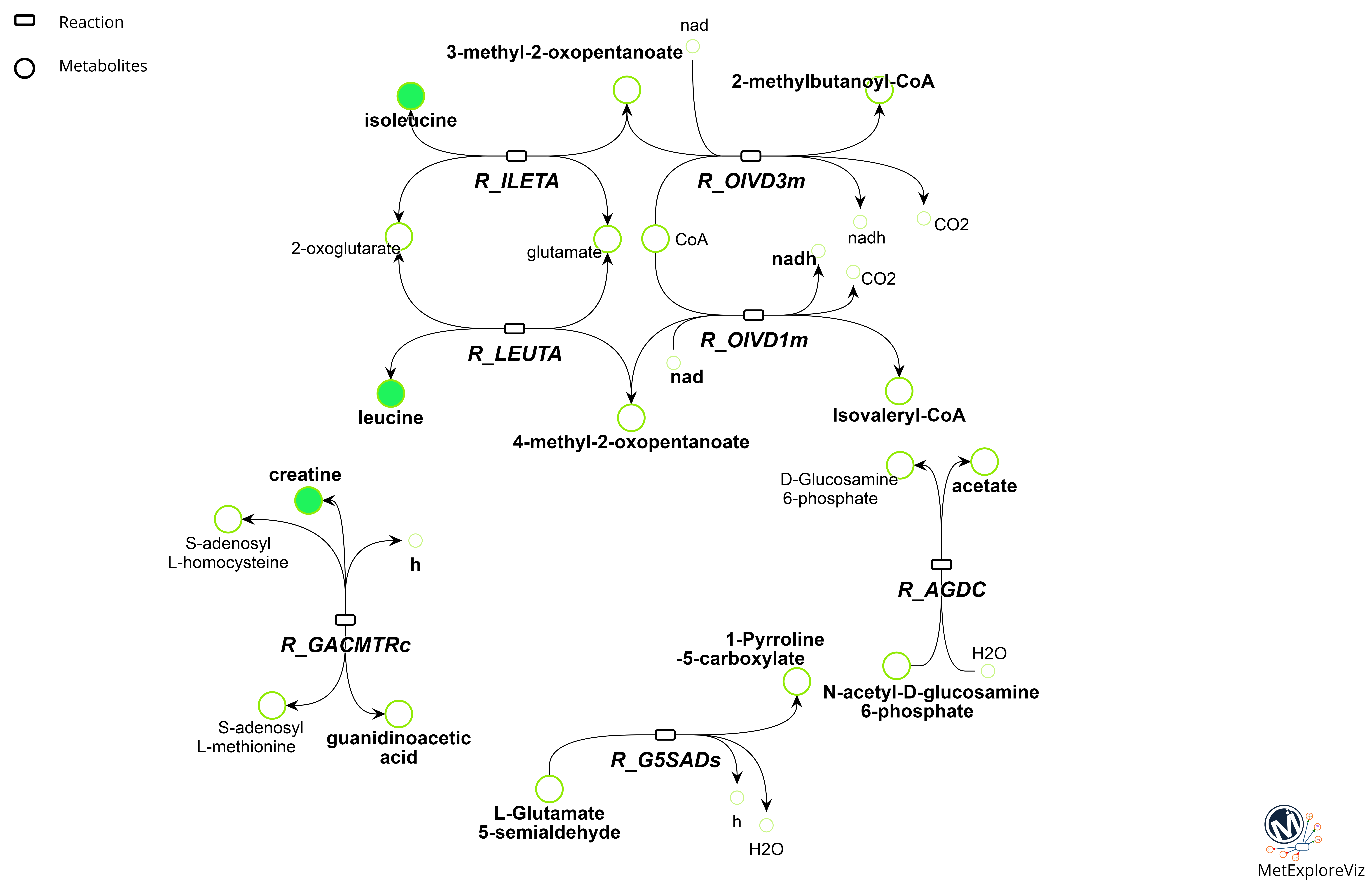

Supplement: Supplementary file 4 [file Image_1.TIF]

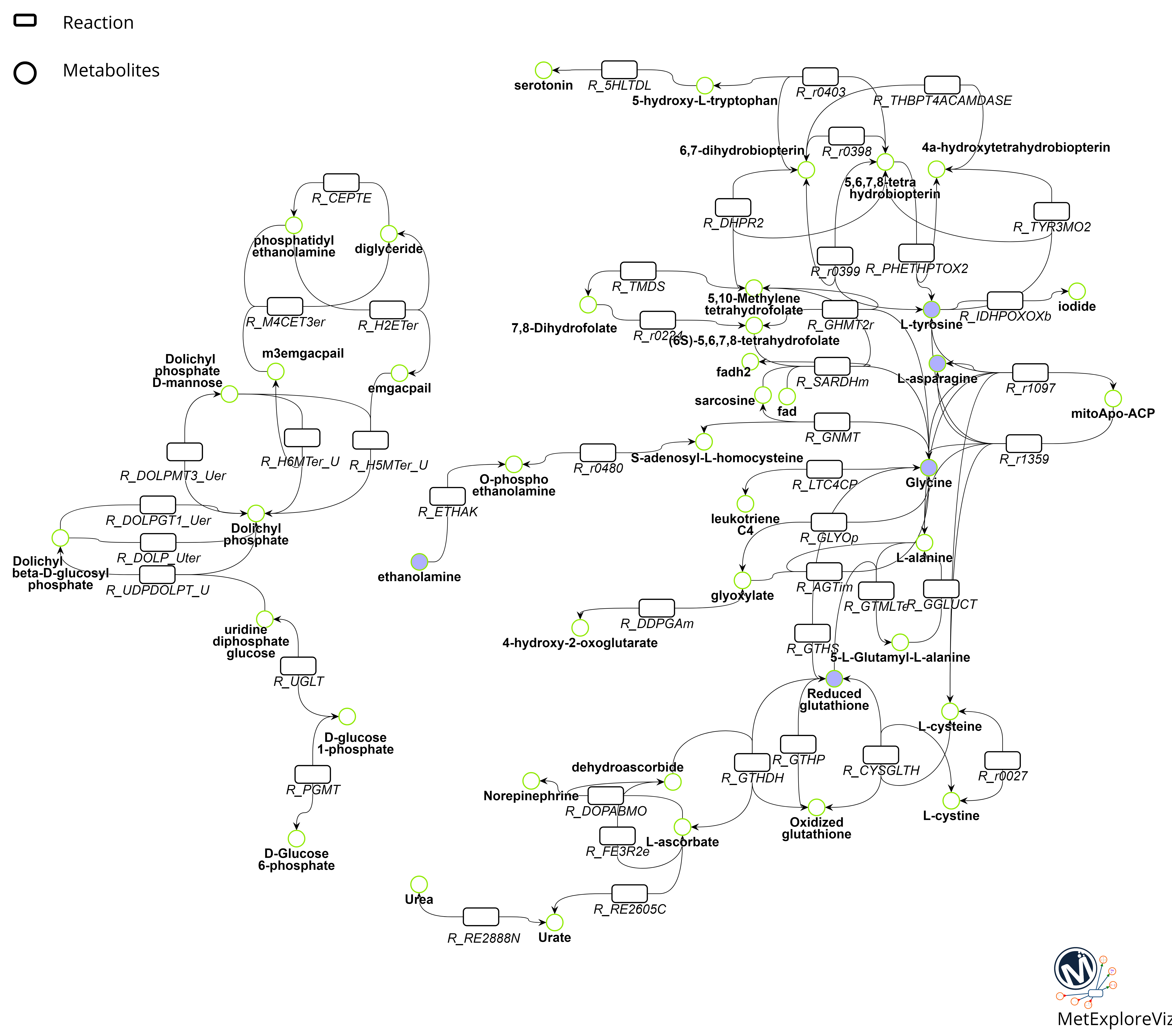

Supplement: Supplementary file 5 [file Image_2.TIF]
